# SMARThealth Pregnancy: Feasibility and Acceptability of a Complex Intervention for High-Risk Pregnant Women in Rural India: Protocol for a Pilot Cluster Randomised Controlled Trial

**DOI:** 10.3389/fgwh.2021.620759

**Published:** 2021-05-28

**Authors:** Shobhana Nagraj, Stephen H. Kennedy, Vivekananda Jha, Robyn Norton, Lisa Hinton, Laurent Billot, Eldho Rajan, Varun Arora, Devarsetty Praveen, Jane E. Hirst

**Affiliations:** ^1^Nuffield Department of Women's & Reproductive Health, University of Oxford, Oxford, United Kingdom; ^2^The George Institute, Imperial College London, London, United Kingdom; ^3^The George Institute for Global Health, New Delhi, India; ^4^University of New South Wales, Kensington, NSW, Australia; ^5^Prasanna School of Public Health, Manipal Academy of Higher Education, Manipal, India; ^6^The George Institute for Global Health, UNSW Sydney, Kensington, NSW, Australia; ^7^THIS Institute (The Healthcare Improvement Studies Institute), Department of Public Health and Primary Care, University of Cambridge, Cambridge, United Kingdom; ^8^Post Graduate Institute of Medical Science, Rohtak, India

**Keywords:** high-risk pregnancy, gestational diabetes, hypertensive disorders of pregnancy, pre-eclampsia, anaemia in pregnancy, cardiometabolic disorders

## Abstract

**Introduction:** India is in the process of a major epidemiological transition towards non-communicable diseases. Cardiovascular disease (CVD) is the leading cause of death in women in India. Predisposing independent risk factors include pregnancy-related conditions, e.g., hypertensive disorders of pregnancy (HDP) and gestational diabetes (GDM) - also associated with significant perinatal mortality and morbidity. Early identification, referral and management of pregnant women at increased risk of future CVD may offer opportunities for prevention. In rural India, Community Health Workers (CHWs) provide most antenatal and postnatal care. Innovative solutions are required to address integrated care for rural women during transitions between antenatal, postnatal and general health services. The George Institute's SMARThealth Programme has shown that CHWs in rural India screening non-pregnant adults for cardiovascular risk, using a decision support system, is feasible. Building on this, we developed a targeted training programme for CHWs and a complex system-level intervention that uses mobile clinical decision support for CHWs and primary care doctors to screen high-risk pregnant women. In addition to addressing HDP and GDM, the intervention also screens for anaemia in pregnancy.

**Methods/Design:** A pilot study will be undertaken in two diverse rural districts of India: Jhajjar (Haryana) and Guntur (Andhra Pradesh). Two Primary Health Centre clusters will be randomised to intervention or control groups at each study site. The primary objective of this pilot study is to explore the feasibility and acceptability of the SMARThealth Pregnancy intervention. Secondary objectives are to estimate: (a) prevalence rates of moderate to severe anaemia, HDPs and GDM at the study sites; (b) referral and follow-up rates, and (c) mean haemoglobin and blood pressure values at the routine 6 week postnatal visit. A process evaluation will be conducted to explore the acceptability of the SMARThealth Pregnancy intervention for pregnant women and healthcare workers using qualitative methods.

**Discussion:** It is anticipated that the findings of this pilot study will help determine the feasibility and acceptability of the SMARThealth Pregnancy intervention, and highlight how the intervention might be further developed for evaluation in a larger, cluster randomised controlled trial.

**Clinical Trial Registration:** www.ClinicalTrials.gov, identifier: NCT03968952.

## Introduction

Cardiovascular disease (CVD) is the leading cause of death in women in India (1), and the prevalence of cardiometabolic disorders is rising (2). Two-thirds of the Indian population live in rural areas (3), where lack of health education and access to health services make women less likely to seek care for cardiometabolic disorders. Pregnancy-related conditions including hypertensive disorders of pregnancy (HDP) and gestational diabetes mellitus (GDM) carry independent risks for future CVD in women (4–8). Early identification, referral and management of pregnant women at increased risk of future cardiometabolic disorders may offer opportunities for prevention. Innovative solutions are required to address the needs of rural women, particularly during the transitions between antenatal and postpartum care and adult health services.

Community Health Workers (CHWs) known as Auxiliary Nurse Midwives (ANMs) and Accredited Social Health Activists (ASHAs) in rural India, provide the majority of antenatal and postnatal care. Task-sharing with CHWs and use of digital and mhealth interventions in rural India have been shown to facilitate the detection, referral and management of non-communicable diseases (NCDs) in non-pregnant adults (9, 10), and HDP in pregnant women (11). There is, however, a paucity of high-quality evidence to guide the postnatal management of women following high-risk pregnancy, including those with multi-morbidity in low-resource settings. Most randomised trials in the postnatal period have focused on neonatal outcomes (12, 13), rather than focus on women - who remain at risk of both immediate complications and future cardiometabolic disorders.

We conducted an in-depth contextual study in rural areas of two diverse states in India (Haryana and Andhra Pradesh) exploring women's and healthcare professionals' understanding of high-risk conditions in pregnancy and their sequalae during and after pregnancy (14). The study highlighted that anaemia in pregnancy was considered as a priority area for high-risk pregnant women by healthcare workers and government officials. We further identified the need for education to improve awareness and institution of standardised screening to identify women with pregnancies complicated by HDP and GDM, and institute a regular follow up schedule. This contextual study provided the foundation for the theory-informed development of a complex intervention (SMARThealth Pregnancy), consisting of mobile clinical decision support, and a targeted education and training programme for CHWs. The focus of the SMARThealth Pregnancy intervention is to address improvement of women's postpartum health, through provision of clinical decision support and training to CHWs in relation to three priority areas: Anaemia, HDP and GDM, during the crucial transition between antenatal and postnatal care, and general health services. The intervention aims to connect pregnant women identified to be at high risk of future cardiometabolic disorders to the Government of India's non-communicable disease (NCD) programme for ongoing, life-long follow-up. The intervention is informed by behaviour change theory (15–17) and the MRC framework for complex interventions (18).

The underlying hypothesis of this pilot study is that the SMARThealth Pregnancy intervention can improve the detection, referral and management of anaemia, HDP, and GDM during the antenatal and postnatal period; and improve knowledge of CHWs to enable them to manage high-risk pregnancies in two areas of rural India. We aim to develop sufficient evidence to design a larger trial that would test the effectiveness of the intervention on an adequate scale.

### Aims and Objectives

The aim of this pilot study is to assess address the feasibility and acceptability of the SMARThealth Pregnancy intervention; determine whether the intervention integrates within the existing health system, and identify any unintended consequences.

The primary objectives of the study are to:

1. Determine whether it is feasible to recruit clusters of Primary Health Centres (PHCs) and pregnant women to the study.
2. Evaluate the intervention's acceptability to end-users (including CHWs and pregnant/postpartum women), to ensure the proposed intervention is feasible and optimised for a definitive trial.

The secondary objectives include:

1. Determine prevalence rates of moderate to severe anaemia, HDP and GDM in pregnant women at the study sites.
2. Determine referral rates for these conditions.
3. Determine loss to follow-up rates up to 6 weeks postpartum.
4. Assess the extent to which the intervention was delivered as planned by CHWs (intervention fidelity) by looking at the completeness of the entries into the mHealth tablet and the timing of these entries.
5. Collection of clinical outcome data, including mean blood pressure (BP) and haemoglobin (Hb) levels at 6–10 weeks postpartum, to inform a sample size calculation and intraclass correlation coefficient (ICC) required for a larger cluster randomised controlled trial (cRCT).
6. To assess CHWs' (ANMs and ASHAs) knowledge retention following a targeted educational programme on anaemia in pregnancy, HDP and GDM, and their long-term sequelae.

## Methods

### Study Design

This is a prospective, parallel, unblinded, cluster randomised controlled pilot study (cRCT). The design was chosen to model and assess study practises including participant recruitment, retention and fidelity of intervention delivery to inform the design of a future definitive trial. The study protocol was developed using the CONSORT 2010 statement extension for randomised pilot and feasibility trials (19). As part of the evaluation process, a qualitative sub-study will be conducted with participants and healthcare workers. This pilot trial was registered with ClinicalTrials.gov (NCT03968952) (20).

### Study Setting

The study will be conducted in rural districts of two states in India: Jhajjar (Haryana) and Guntur (Andhra Pradesh). Haryana is a state in northern India, whose history of significant gender inequity (21), has improved over the last decade due to targeted legislation and policies, as well as community-based action groups. Andhra Pradesh is a state in south-eastern India with high rates of diabetes and hypertension in the adult population (22). The two states are geographically and culturally diverse, with unique languages, cuisine and practises. The maternal mortality ratio is 101/100,000 births in Haryana, and 74/100,000 births in Andhra Pradesh (23). In the National Family Health Survey IV (24), total prevalence of hypertension and diabetes in women of reproductive age was 10.1% in Andhra Pradesh, and 9.2% in Haryana. Prevalence of raised blood sugar indicative of diabetes in women of reproductive age in Andhra Pradesh and Haryana was 13.1 and 6.6%, respectively (24).

### Sampling

A list of PHCs in each district will be compiled using data from Government of India State websites (25, 26). The PHCs will be stratified by geographic region and population size (each PHC to serve a population above 30,000) to include: up to eight primary care doctors (two at each PHC cluster), up to 20 ANMs (five at each cluster), and 80 ASHAs (20 at each cluster). The villages selected under each intervention and control PHC will be non-contiguous to avoid contamination. Each PHC will be assessed for eligibility (see below). Consent for the PHC and its staff to participate in the study will be obtained from the administrative lead at each eligible PHC prior to randomisation. Please see Table 1 for the study inclusion criteria.

**Table 1:** Summary of inclusion criteria for the study.

**Inclusion criteria** • Primary Health Centres (PHCs) serving a population of 30,000 people or more, in either the: (a) Jhajjar district of Haryana or (b) Guntur district of Andhra Pradesh. • Primary care doctors working at the participating PHCs, in either (a) Jhajjar district of Haryana or (b) Guntur district of Andhra Pradesh. • Community Health Workers (ANMs and ASHAs) working in partnership with the participating PHCs, in either (a) Jhajjar district of Haryana or (b) Guntur district of Andhra Pradesh. • Pregnant women above the age of 18 years (adults) living within the villages in the catchment area of the PHCs participating in the study, in either (a) Jhajjar district of Haryana or (b) Guntur district of Andhra Pradesh. ^*^PHCs and participants not meeting the above criteria will be excluded from the study

### Randomisation

Randomisation will be at the PHC cluster level, as individual randomisation would not be possible due to potential contamination. Four PHCs (including the villages affiliated to each PHC) will be allocated centrally to the SMARThealth Pregnancy intervention or control group (enhanced standard care), using a simple random number generator. This process will be overseen by a statistician at The George Institute for Global Health (TGIGH), blinded to the PHCs.

### Participants

We will recruit 200 pregnant women (50 in each of the four PHC clusters) from the two districts. Participants will be ≥18 years old (adults), between 28 and 36 weeks' gestation, and followed up until and including 6 weeks postpartum. The last trimester of pregnancy was chosen to recruit participants as the contextual study (14) revealed women migrated to their mother's homes prior to delivery. We wanted to ensure these women were connected to health services after migration and received postpartum follow-up. After discussion with a statistician at TGIGH (LB), four clusters from two different districts recruiting a total of 200 pregnant women were considered pragmatic to provide rich feasibility data, as well as early estimates of clinical outcomes. As the focus of this pilot study is to assess the feasibility and acceptability of conducting a definitive trial, no formal sample size calculation was undertaken.

### Recruitment and Consent

Eligible pregnant women will be identified by the ANM during their standard antenatal care visits. The ANM will inform the locally-based research assistant from TGIGH, India, of eligible participants. The research assistant will then obtain informed consent to participate in the study.

**Figure 1:**
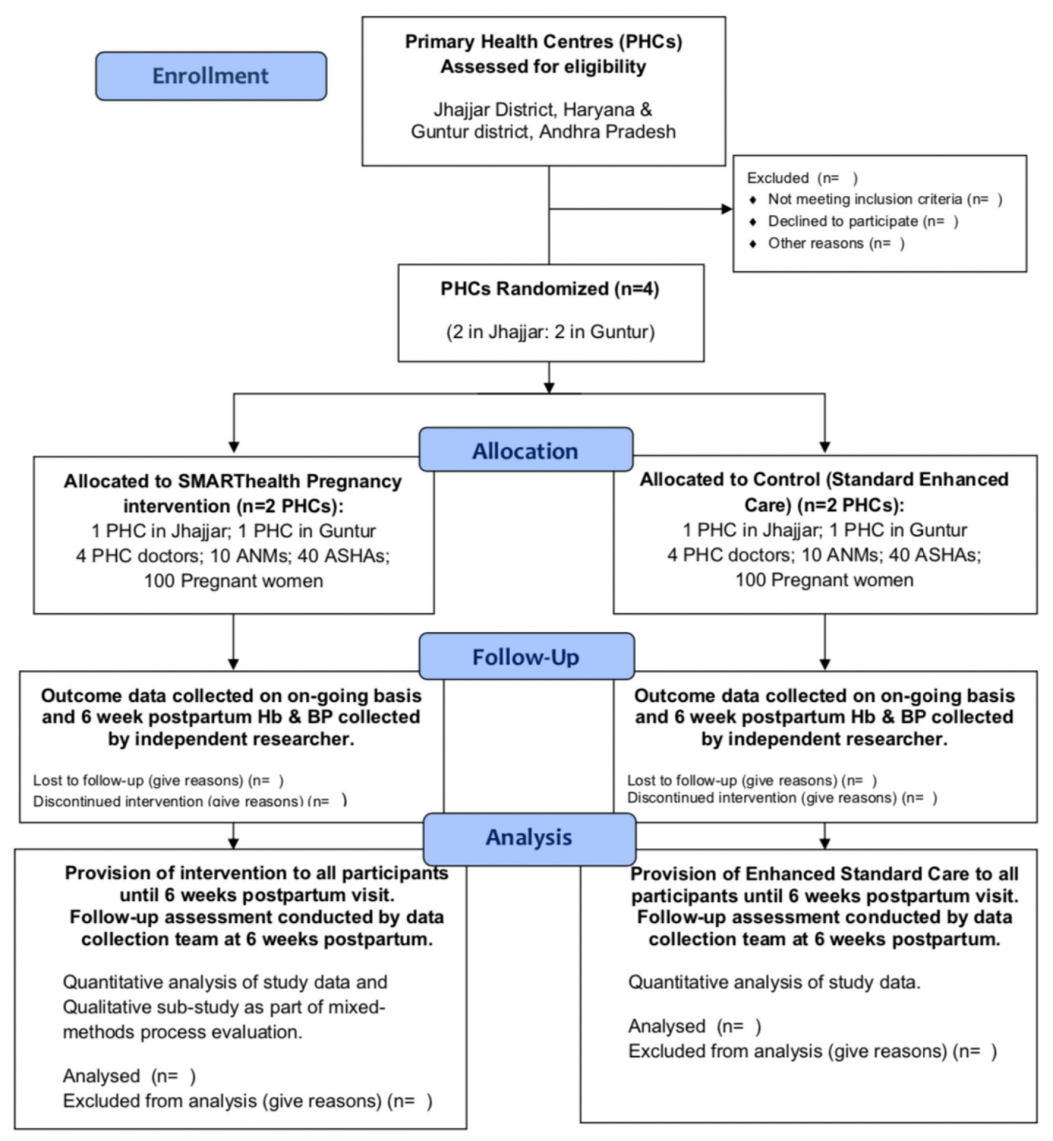
Participant flowchart of pilot study.

Women in the intervention group will be asked to consent to having their BP and Hb measured by an ASHA at three time points during: (a) the last trimester of pregnancy (between 28 and 36 weeks gestation); (b) week 1 postpartum and (c) week 6 postpartum. They will also consent to sharing their antenatal care handheld record containing their demographic details, medical and obstetric history, and details of all their previous BP and Hb readings. In addition, they will consent to having their BP and Hb measured at home by an independent member of the research team at the baseline of the study, and after the final 6 week postpartum visit (see Figure 1).

Women in the control group will be asked to consent to sharing their antenatal care handheld record, containing their demographic details, medical and obstetric history, and details of all their previous BP and Hb readings from their antenatal visits. In addition, they will consent to having their BP and Hb measured at home by an independent member of the research team at the baseline of the study, and after their routine 6 week postpartum visit. Individual consent will be sought from CHWs and doctors prior to interviews and FGDs.

## The SMARThealth Pregnancy intervention

The SMARThealth Pregnancy intervention includes two components:

1. A targeted educational and training component for CHWs on anaemia in pregnancy, HDP and GDM.
2. An mHealth platform providing clinical decision support and lifestyle advice for ASHAs and primary care doctors.

**Figure 2:**
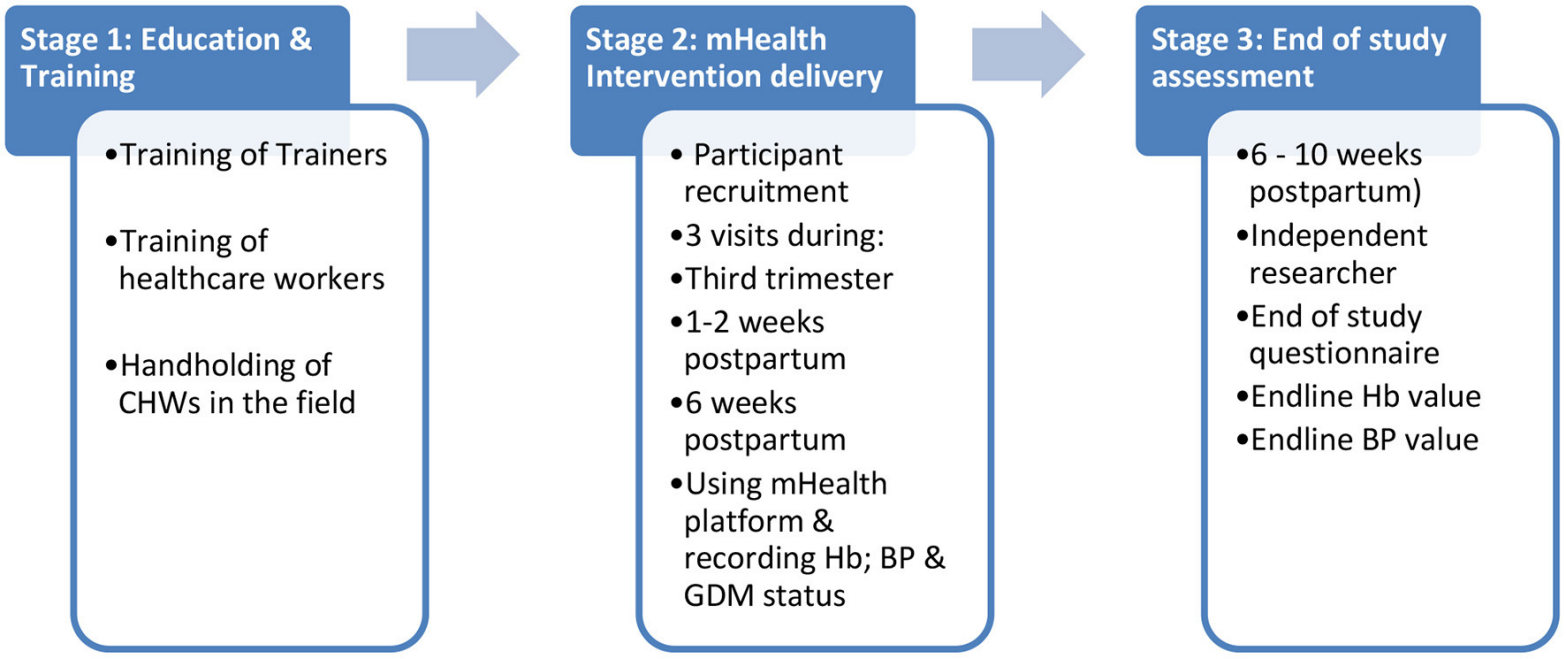
Stages in SMARThealth pregnancy study.

The intervention will be delivered in three stages: (1) Education and Training; (2) Delivery of the mHealth Intervention and (3) End of study assessment (see Figure 2).

### Stage 1: Education and Training

A train the trainers programme has been developed for all study staff on the importance of identifying women with a high-risk pregnancy; detecting anaemia, HDP and GDM antenatal and postnatally, and the impact of these conditions on the life-long health of women. The programme also outlines the delivery of the standardised training for CHWs.

The training curriculum for CHWs has been designed to build upon existing CHW training on high-risk pregnancies, and cover key learning objectives in relation to the importance of identification of high-risk pregnant women, with a focus on detection, referral and management of HDP, GDM, and anaemia in pregnancy according to established clinical guidelines (27–31). CHWs will also be taught how to use the SMARThealth Pregnancy mHealth mobile tablet, and provided information on what to do if the technology does not work properly. They will additionally learn the following skills:

1. Measuring BP using the CRADLE VSA Blood Pressure Monitor (APEC, UK) (32) and interpreting the readings. The CRADLE device is a low-cost semi-automated BP device, specifically designed for low resource settings, charged using an android phone charger, and has been used by CHWs in India (31).
2. Measuring Hb using the handheld TrueHb Hemometer (Wrig nanosystems Pvt. Ltd., 2020) (33) and interpretation of readings.
3. Communication of findings and concerns by ASHA to ANM/primary care doctor, and skills in handover of care between health professionals.

Training will be delivered to ASHAs and ANMs in a series of five participatory learning sessions each of 3-h duration conducted over two and a half days, with a 1-day face-to-face refresher training session after 4 weeks. In between the two training sessions, there will be “hand-holding” in the field by the local study field team, to ensure the ASHAs feel confident using the blood pressure device, haemoglobinometer and the tablet device (see below).

The curriculum for primary care doctors will cover the same areas as for CHWs, however the delivery will be over a half-day of one-to-one training, assuming a degree of baseline knowledge on both educational and training components.

### Stage 2: The SMARThealth Pregnancy mHealth Platform

The SMARThealth Pregnancy mHealth platform is a smartphone-based application, using an Android operating system, on a seven inch mobile tablet. The application is multi-faceted and includes provision of clinical decision support for ASHAs and primary care doctors regarding interpretation of BP, Hb and blood sugar measurements, grading of severity and appropriate referral information using established clinical guidelines (27–31). Mobile tablets will be provided to ASHAs working in the villages, and to the primary care doctor at the corresponding PHC.

In addition to standard antenatal and postnatal visits, ASHAs in the intervention group will visit the pregnant woman enrolled in the study at home, and perform BP readings, and take a finger-prick Hb test at three different time points:

1. Once during the last trimester of pregnancy between 28 and 36 weeks' gestation.
2. At 1–2 weeks postpartum
3. At 6 weeks postpartum

The SMARThealth Pregnancy mHealth platform will have options for the ASHAs to enter the input measurements to generate recommendations based on the woman's history, BP, Hb and blood glucose readings, which will be confidentially uploaded to a patient record using Open MRS software (34) and synchronise to a sister-tablet held by the primary care doctor at the local PHC. All recommendations are based on established country-specific clinical guidelines. Women will be referred to the doctor at the local PHC via the mHealth platform, and provided with a paper referral card. Although women cannot be compelled to see the primary care doctor, they will however be encouraged to attend the PHC after each ASHA visit. The ASHA will accompany the woman for the visit to the primary care doctor or to secondary care as per current standard practise.

Through the SMARThealth Pregnancy mHealth platform, the primary care doctor will be able to note if the woman is anaemic (and the severity), hypertensive, and/or screen-positive for GDM. The primary care doctor will be guided to repeat these readings if the patient attends the PHC, with access to guideline-based decision support for anaemia, hypertension and GDM as to the next steps in management/and or referral to secondary care. Priority patients (identified to have high-risk pregnancy conditions, and/or outstanding investigations), will have a red traffic light alert and be automatically placed at the top of the priority list when the SMARThealth Pregnancy application is launched on the ASHA and primary care doctor tablets.

Postnatal visits take the same course as the antenatal visit, except the history-taking will involve details of the birth and labour (Postpartum: Week 1 visit), and any further complications (Postpartum: Week 6 visit).

To ensure quality assurance for intervention practises, regular field visits and observations of ASHAs and primary care doctors will be made by the research team and project managers at each study site to ensure compliance to study procedures and the fidelity of the intervention practises. In addition, data collected through the mHealth tablets will be used to monitor visits made and completed by ASHAs and primary care doctors.

### Control Group

The control group will receive *enhanced standard* antenatal and postnatal care, involving: An awareness programme for pregnant women, CHWs and primary care doctors, conducted during an established village health and nutrition day camp (held monthly), at the villages within the control group PHC cluster. The community and health professionals will receive information on the high-risk conditions of anaemia in pregnancy, HDP and GDM as part of the awareness programme.

### Stage 3: End of Study Assessment

After the final 6 week postpartum ASHA visit, an independent researcher (not blinded to the intervention) will visit the woman at home and complete an end of study questionnaire, and record BP and Hb readings independently in both the intervention and control groups. This visit will be conducted between 6 and 10 weeks postpartum allowing for convenience of study participants. They will also document if women in the control group had received a 6 week postpartum visit from their ASHA/ANM.

### Primary and Secondary Outcomes

Primary outcomes relating to feasibility of the intervention include:

1. Recruitment rates of pregnant participants per cluster per month.
2. Retention rates of participants at 6 weeks postpartum.
3. An overview of the acceptability of the SMARThealth Pregnancy intervention using a qualitative sub-study.

Secondary outcomes include:

1. Prevalence rates of moderate to severe anaemia, HDP and GDM diagnosed in the study sites.
2. Assessment of fidelity of the intervention by:
  - Proportion of visits conducted in line with intervention protocol.
  - Proportion of visits completed by ASHA (including measurement of Hb and BP) at visits during: (a) third trimester, (b) 1–2 weeks postpartum, (c) 6 weeks postpartum, in the intervention group.
  - Proportion of pregnant women detected, referred, managed, and followed-up, with moderate to severe anaemia, HDP or GDM.
3. Proportion of women receiving their 6 week postnatal check by the ASHA.
4. Clinical outcomes: Mean Hb and mean BP at 6 week postpartum visit.

### Process Evaluation

As part of the process evaluation, a qualitative sub-study will be conducted to explore the acceptability of the SMARThealth pregnancy intervention to healthcare workers and pregnant/postpartum women; whether the intervention components had been delivered to plan; how the intervention integrates into existing health system practises; any unintended consequences; and to understand how to improve and refine the intervention and its implementation. A purposive sample of participants including pregnant/postpartum women, CHWs and primary care doctors from the intervention groups at each study site will be invited to share their experiences of the SMARThealth Pregnancy intervention in focus group discussions (FGDs) and/or in-depth interviews (IDIs). We aim to conduct up to four FGDs involving pregnant/postpartum women; up to four FGDs with CHW, and up to four IDIs with primary care doctors across both study sites. Interviews and focus groups will be conducted with CHWs and pregnant/postpartum women at regular intervals during the study: after they have conducted/received visit 1, visit 2 and visit 3; to assess acceptability of each visit to participants as the study progresses. IDIs will be held with doctors at the end of the study to minimise inconvenience due to the workforce constraints affecting primary care doctors in rural areas. Qualitative data from the process evaluation will be analysed using a framework approach (35) using nVIVO QSR^©^ software to facilitate the organisation of the data into codes and categories.

### Statistical Analysis

Analysis of the pilot study will focus on descriptive statistics rather than formal hypothesis testing. Quantitative data relating to the detection, referral, treatment and follow-up rates of pregnant women with (a) anaemia; (b) HDPs; and (c) GDM will be collected for the intervention group. Data relating to the number of pregnant women participating in the study, will allow the proportions of women with these conditions, receiving treatment and follow-up care to be calculated. The proportion of women having their 6 week postpartum visit will also be calculated in both groups. The feasibility of the recruitment strategy and the retention of participants throughout the study will be summarised. Statistical analysis will be conducted at the end of the study once all data collection is completed.

Even though this is a pilot study, we will explore for any differences between the intervention and treatment groups, using an intention-to-treat analysis. Mean differences in 6 week postpartum Hb and BP will be calculated using an analysis of covariance adjusted for the baseline outcome value and for clustering by site. All statistical analysis will be performed using the Statistical Programme for Social Sciences (SPSS^©^).

To inform the sample size calculation for a larger cluster randomised controlled trial, we will calculate the standard deviation around the mean Hb and BP values at 6 weeks postpartum as well as the intra-cluster correlation (ICC) and its 95% confidence interval. ICC estimates will be derived from a mixed-effect model using SAS software^©^.

### Data Monitoring and Confidentiality

Data generated through the SMARThealth Pregnancy mHealth platform will be securely uploaded (using Open MRS v1.9) and stored on a secure server at The George Institute, India. All data will be de-identified and anonymised. Data will be monitored for quality and completeness by the programme manager at regular intervals as it is uploaded to the server. All data will be stored and handled in accordance with the principles of the 1998 Data Protection Act.

### Ethical Approval and Trial Oversight

Ethical approval for the study has been obtained from the Oxford Tropical Research Ethics Committee (OxTREC reference: 22-19) and the George Institute India Ethics Committee (Reference: 010/2019).

The trial steering committee will oversee the study and be responsible for participant safety. Regular operation group meetings and field team meetings will be held with relevant members of the team. The pilot study sponsor is the University of Oxford, and the study will be funded by the George Institute for Global Health India, and the Medical Research Council (individual MRC fellowship to SN: MR/R017182/1).

## Discussion

The importance of addressing the rising burden of cardiometabolic disorders in women in rapidly developing low- and middle-income countries, such as India, is becoming a priority. Early detection of at-risk women during pregnancy and implementing appropriate interventions might be one way to alleviate this burden (36). Studies have shown that almost half of Indian women with GDM, convert to type 2 diabetes within 5 years postpartum (37, 38), providing a key window for prevention. HDP are also associated with an increased risk of future CVD (39, 40), although the trajectory is longer. There is uncertainty as to the best time to counsel women regarding their long-term health, and what evidence-based strategies might be successfully implemented following pregnancy in high-risk women. Balancing the long term needs of women and their healthcare priorities during pregnancy (such as anaemia), requires careful thought. The SMARThealth Pregnancy intervention seeks to introduce concepts of integrated life-long women's health into standard antenatal and postnatal care, whilst strengthening existing practices. There is evidence to suggest that involving CHWs in women's health is feasible (41) and can improve antenatal care practises (42, 43), although no CHW-led intervention studies focusing exclusively on high-risk pregnancy have to date shown an improvement on clinical endpoints (43, 44). Additionally, the lack of evidence regarding the effectiveness of mHealth solutions in improving clinical outcomes in LMIC settings is an important barrier to their wide use (45). This pilot cRCT seeks to provide sufficient feasibility data to enable refinement of the SMARThealth Pregnancy mHealth intervention and to model future trial processes. The results of this study will build upon the existing evidence for CHW-led community-based digital interventions for women, as well as extending this evidence to include ongoing postpartum follow-up; connecting high-risk women to local NCD programmes, and using antenatal care as a window for engagement with the health system. The study will further identify important factors for strengthening primary care in rural India in relation to providing an integrated life-course approach to women's health.

## Conclusion

This protocol has outlined the aims and objectives of a pilot cRCT of a complex intervention, with a detailed overview of the intervention components and delivery. The pilot study will impact on the decision to conduct a larger cRCT and will help refine the intervention and trial practises. It will add to the evidence for the feasibility of early detection of high-risk women, and CHW-led community-level interventions and strategies for addressing pregnancy-related conditions and life-long women's health.

## Author Contributions

SN designed the pilot study protocol and wrote the first draft of the article. SK, VJ, RN, LH, LB, ER, VA, DP, and JH contributed to editing the draft for publication. All authors contributed to the article and approved the submitted version.

## Conflict of Interest

The authors declare that the research was conducted in the absence of any commercial or financial relationships that could be construed as a potential conflict of interest.
